# GR-891: a novel 5-fluorouracil acyclonucleoside prodrug for differentiation therapy in rhabdomyosarcoma cells

**DOI:** 10.1038/sj.bjc.6690129

**Published:** 1999-02

**Authors:** J A Marchal, J Prados, C Melguizo, J A Gómez, J Campos, M A Gallo, A Espinosa, N Arena, A Aránega

**Affiliations:** Departamento de Ciencias Morfológicas, Facultad de Medicina, Universidad de Granada, Granada, E-18071, Spain; Departamento de Ciencias de la Salud y Psicología Clínica, Universidad de Almería, Almería, E-23071, Spain; Departamento de Química Orgánica, Facultad de Farmacia, Universidad de Granada, Granada, E-18071, Spain; Istituto di Istologia, Facultá di Medicina e Chirurgia, Università di Sassari, Sassari, 07100, Italy

**Keywords:** acyclonucleoside prodrugs, 5-fluorouracil, differentiation therapy, multidrug resistance, rhabdomyosarcoma

## Abstract

Differentiation therapy provides an alternative treatment of cancer that overcomes the undesirable effects of classical chemotherapy, i.e. cytotoxicity and resistance to drugs. This new approach to cancer therapy focuses on the development of specific agents designed to selectively engage the process of terminal differentiation, leading to the elimination of tumorigenic cells and recovery of normal cell homeostasis. A series of new anti-cancer pyrimidine acyclonucleoside-like compounds were designed and synthesized by structural modifications of 5-fluorouracil, a drug which causes considerable cell toxicity and morbidity, and we evaluated their applicability for differentiation therapy in human rhabdomyosarcoma cells. We tested the pyrimidine derivative GR-891, (RS)-1-{[3-(2-hydroxyethoxy)-1-*iso*propoxy]propyl}-5-fluorouracil, an active drug which shows low toxicity in vivo and releases acrolein which is an aldehyde with anti-tumour activity. Both GR-891 and 5-fluorouracil caused time- and dose-dependent growth inhibition in vitro; however, GR-891 showed no cytotoxicity at low doses (22.5 μmol l^−1^ and 45 μmol l^−1^) and induced terminal myogenic differentiation in RD cells (a rhabdomyosarcoma cell line) treated for 6 days. Changes in morphological features and in protein organization indicated re-entry in the pathway of muscular maturation. Moreover, GR-891 increased adhesion capability mediated by the expression of fibronectin, and did not induce overexpression of P-glycoprotein, the *mdr1* gene product, implicated in multidrug resistance. New acyclonucleoside-like compounds such as GR-891 have important potential advantages over 5-fluorouracil because of their lower toxicity and their ability to induce myogenic differentiation in rhabdomyosarcoma cells. Our results suggest that this drug may be useful for differentiation therapy in this type of tumour. *1999 Cancer Research Campaign*


*British Journal of Cancer* (1999) **79**(5/6), 807–813
© 1999 Cancer Research Campaign
Article no. bjoc.1998.0129

### GR-891: a novel 5-fluorouracil acyclonucleoside prodrug for differentiation therapy in rhabdomyosarcoma cells

**JA Marchal[1], J Prados[2], C Melguizo[2], JA Gómez[3], J Campos[3], MA Gallo[3], A Espinosa[3], N Arena[4] and A Aránega[1]**

[1]Departamento de Ciencias Morfológicas, Facultad de Medicina, Universidad de Granada, E-18071 Granada, Spain; [2]Departamento de Ciencias de la Salud y Psicología Clínica, Universidad de Almería, E-23071 Almería, Spain; [3]Departamento de Química Orgánica, Facultad de Farmacia, Universidad de Granada, E-18071 Granada, Spain; and [4]Istituto di Istologia, Facultá di Medicina e Chirurgia, Università di Sassari, 07100 Sassari, Italy

**Summary** Differentiation therapy provides an alternative treatment of cancer that overcomes the undesirable effects of classical chemotherapy, i.e. cytotoxicity and resistance to drugs. This new approach to cancer therapy focuses on the development of specific agents designed to selectively engage the process of terminal differentiation, leading to the elimination of tumorigenic cells and recovery of normal cell homeostasis. A series of new anti-cancer pyrimidine acyclonucleoside-like compounds were designed and synthesized by structural modifications of 5-fluorouracil, a drug which causes considerable cell toxicity and morbidity, and we evaluated their applicability for differentiation therapy in human rhabdomyosarcoma cells. We tested the pyrimidine derivative GR-891, (RS)-1-{[3-(2-hydroxyethoxy)-1-*iso*propoxy]propyl}-5-fluorouracil, an active drug which shows low toxicity in vivo and releases acrolein which is an aldehyde with anti-tumour activity. Both GR-891 and 5-fluorouracil caused time- and dose-dependent growth inhibition in vitro; however, GR-891 showed no cytotoxicity at low doses (22.5 $\mu$mol $l^{-1}$ and 45 $\mu$mol $l^{-1}$) and induced terminal myogenic differentiation in RD cells (a rhabdomyosarcoma cell line) treated for 6 days. Changes in morphological features and in protein organization indicated re-entry in the pathway of muscular maturation. Moreover, GR-891 increased adhesion capability mediated by the expression of fibronectin, and did not induce overexpression of P-glycoprotein, the *mdr1* gene product, implicated in multidrug resistance. New acyclonucleoside-like compounds such as GR-891 have important potential advantages over 5-fluorouracil because of their lower toxicity and their ability to induce myogenic differentiation in rhabdomyosarcoma cells. Our results suggest that this drug may be useful for differentiation therapy in this type of tumour.

**Keywords:** acyclonucleoside prodrugs; 5-fluorouracil; differentiation therapy; multidrug resistance; rhabdomyosarcoma

The goal of cancer chemotherapy with classical drugs – the destruction of the tumour cells – is often complicated by significant toxicity. Differentiation therapy is a new approach to cancer treatment which assumes that neoplastic transformation reveals the inability of a cell population to couple proliferation and differentiation signals. Induced differentiation modulates the cell programme by transforming malignant cells into mature cells with no proliferative potential (Sartorelli, 1985; Borden et al, 1993). Cells mature, become differentiated, die, disappear and are replaced by normal cells. The addition of differentiation treatment to chemotherapy greatly improves the chance of long-term survival (Degos, 1995). However, the development of multidrug resistance (MDR) phenotype in tumour cells during treatment can be the major obstacle in the differentiation therapy of cancer. Although standard agents used in the treatment of tumours may induce several mechanisms of drug resistance, classic MDR has been related to the overexpression of the *mdr1* gene, which encodes P-glycoprotein. This protein, which acts as a drug efflux pump, decreases the cellular accumulation of cytotoxic drug (Germann, 1996; Skach, 1996).

*Received 3 March 1998*
*Revised 21 May 1998*
*Accepted 24 July 1998*

*Correspondence to:* A Aránega, Sección de Investigación Básica Cardiovascular, Depto. de Ciencias Morfológicas, Facultad de Medicina, Universidad de Granada, E-18071 Granada, Spain

New differentiative drugs developed from well-known chemical structures endeavour to overcome the two main problems associated with classic chemotherapy: toxicity and the appearance of resistance. Recent years have seen the development of prodrugs designed to induce differentiation in various types of neoplasms (Chen and Breitman, 1994). 5-Fluorouracil (5-FU) in combination with other differentiating agents (Zupi et al, 1988) is able to induce differentiation in tumour cells derived from human colon carcinoma and leukaemia (Waxman et al, 1990, 1992; Lesuffleur et al, 1991*a*,*b*). However, 5-FU is highly toxic to both tumour and normal cells, and it is therefore essential to search for derivatives that mimic the action of 5-FU without producing undesirable side-effects. The pyrimidine acyclonucleoside-like compound (R,S)-1-{[3-(2-hydroxyethoxy)-1-*iso*propoxy]propyl}-5-fluorouracil (GR-891) is a 5-FU acyclonucleoside-like prodrug that shows little or no toxicity in vivo compared with 5-FU (Campos et al, 1996) (Figure 1).

Studies in rhabdomyosarcoma tumours, the most frequent soft tissue malignancy in paediatric patients, have shown that the malignant phenotype can be repressed to some degree, and that the tumour cells can be induced to re-enter the differentiation process (Gabbert et al, 1988; Aguanno et al, 1990; Crouch and Helman, 1991; De Giovanni et al, 1993). Few studies have been published on drug-induced myogenic differentiation in rhabdomyosarcoma chemotherapy (Lollini et al, 1989; Crouch et al, 1993; D'Amore et al, 1994; Melguizo et al, 1995, 1996; Marchal et al, 1997). Other pyrimidine analogues such as cytarabine arabinoside (Crouch et al,

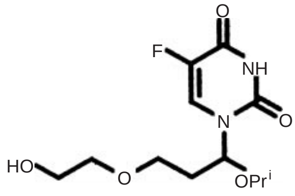

**Figure 1** Structure of (RS)-1-{[3-(2-hydroxyethoxy)-1-*iso*propoxy]propyl}-5-fluorouracil) (GR-891)

1993) have recently been investigated as differentiating drugs for rhabdomyosarcoma. Previously, we showed that these new 5-FU acyclonucleosides are capable of inducing differentiation in rhabdomyosarcoma cells (Gómez et al, 1997). These experiences suggest that this type of tumour may be amenable to treatment by differentiation therapy.

The aim of this study was to evaluate the potential role of GR-891, a new pyrimidine acyclonucleoside-like compound, against rhabdomyosarcomas. Our data demonstrate that GR-891 inhibits proliferation, induces myogenic differentiation, increases the expression of proteins specifically present in normally differentiated skeletal muscle cells, and modifies the adhesion capacity of these cells. In addition, this drug causes no modifications on *mdr1* gene expression of RD cells.

### MATERIALS AND METHODS

### Cell culture

The rhabdomyosarcoma cell line RD (MacAllister et al, 1969), derived from a human embryonic rhadomyosarcoma, was obtained from the American Type Culture Collection (ATCC, Rockville, MD, USA). RD cells were routinely maintained in monolayer cultures at 37°C in an atmosphere containing 5% carbon dioxide with Dulbecco's modified Eagle medium (DMEM) (Gibco, NY, USA) supplemented with 10% fetal bovine serum (FBS) (Gibco), 20 mmol $l^{-1}$ L-glutamine, 3.5 mg $\mu l^{-1}$ sodium bicarbonate, 4.5 g $l^{-1}$ glucose, 250 U $ml^{-1}$ ampicillin and 20 $\mu g$ $ml^{-1}$ streptomycin. The same batch of FBS was used for all experiments to obviate variations in quality.

### Drugs and drug treatments

After synthesis and purification of GR-891 (Campos et al, 1996), stock solutions of GR-891 and 5-FU were prepared. The drugs were dissolved in distilled water, sterilized by filtration, and stored at –20°C. For each experiment, the stock solution was further diluted in medium to the desired concentration. The cells were detached from the surface of the tissue culture flasks with phosphate-buffered saline (PBS)/EDTA (0.02%) and diluted in Dulbecco's medium to obtain cultures of $5 \times 10^6$ cells. RD cells were exposed continuously to three different concentrations of GR-891 and 5-FU: 22.5 $\mu$mol $l^{-1}$, 45 $\mu$mol $l^{-1}$ and 90 $\mu$mol $l^{-1}$. Parallel cultures of RD cells in medium without drugs were used as controls. The medium in both control and drug-treated cultures was replaced every 48 h, and the cultures were maintained and examined every 24 h for 6 days. Separate RD cell cultures were treated with 22.5 $\mu$mol $l^{-1}$, 45 $\mu$mol $l^{-1}$ and 90 $\mu$mol $l^{-1}$ for 3 days, after which GR-891 and 5-FU were removed from the culture media. These cells were allowed to recover log phase growth in normal standard media and were then retreated with the drugs for 6 days.

### Assessment of growth properties in vitro

Eighteen replicate culture flasks (25 $cm^2$) (Greiner, Nürtingen, Germany) containing parental RD cells ($1 \times 10^6$) were exposed to growth medium supplemented with 22.5 $\mu$mol $l^{-1}$, 45 $\mu$mol $l^{-1}$ or 90 $\mu$mol $l^{-1}$ of GR-891 or 5-FU. As a control, culture flasks were seeded with cells in standard growth medium. In each experiment, cells from the culture flasks with each different drug concentration, and three flasks with cells growing in drug-free medium, were harvested separately every 24 h for 6 days. Cell viability was assayed by the trypan blue exclusion test. The number of cells harvested was determined in a model ZBI Kontron Coulter counter.

### In vitro morphology

RD cells were observed with an inverted light microscope and a transmission electron microscope before and after treatment with GR-891 or 5-FU at a concentration of 22.5 $\mu$mol $l^{-1}$, 45 $\mu$mol $l^{-1}$ or 90 $\mu$mol $l^{-1}$. Optic phase-contrast photographs were taken with a Nikon TM Phase Contrast-2, ELWD 0.3 inverted microscope. The number of myotube-like giant cells was counted by phase-contrast microscopy at intervals of 24 h. Cells that contained three or more nuclei were classified as myotube-like giant cells. For transmission electron microscopy, RD cells were treated according to Melguizo et al (1995) and ultrathin sections were cut parallel and perpendicular to the surface of the flask. The sections were contrasted with uranyl acetate/lead citrate and examined in a Hitachi H 7000 transmission electron microscope (TEM).

Adherent tumour cells on coverslips were fixed for scanning electron microscopy (SEM) with 2.0% glutaraldehyde, dehydrated in graded concentrations of ethanol, and dried using the critical point method. These preparations were coated with platinum and observed with a Hitachi S-800 scanning electron microscope.

### Fluorescence-activated cell sorting (FACScan)

Non-treated cells and RD cells treated for 6 days were prepared for FACScan as described elsewhere (Melguizo et al, 1996). The cells were incubated for 30 min at 4°C with the anti-desmin (dilution 1:200) and anti-vimentin (dilution 1:200) monoclonal antibodies (mAbs) (Sigma, St Louis, MO, USA), then washed twice in cold PBS and reincubated with fluorescein isothiocyanate (FITC)-conjugated anti-mouse IgG (Sigma) at a dilution of 1:50 for FACS analysis.

### Immunofluorescent cytochemistry

Cultured RD cells were analysed after 6 days of treatment with 22.5 $\mu$mol $l^{-1}$, 45 $\mu$mol $l^{-1}$ or 90 $\mu$mol $l^{-1}$ of GR-891 and 5-FU. The cells were washed several times with PBS at pH 7.2, fixed with methanol at 4°C for 10 min, washed three times in PBS and incubated with EA-53 anti-$\alpha$-actinin mAb (dilution 1:200) (Sigma) and FN-15 anti-fibronectin mAb (dilution 1:200) (Sigma) at 37°C for 15 min in a humidified chamber. The first incubation was followed by washing four times in PBS and a second incubation at 37°C in a dark, humidified chamber with a 1:50 dilution of FITC-conjugated goat anti-mouse IgG (Sigma) during 30 min. After a

 © Cancer Research Campaign 1999

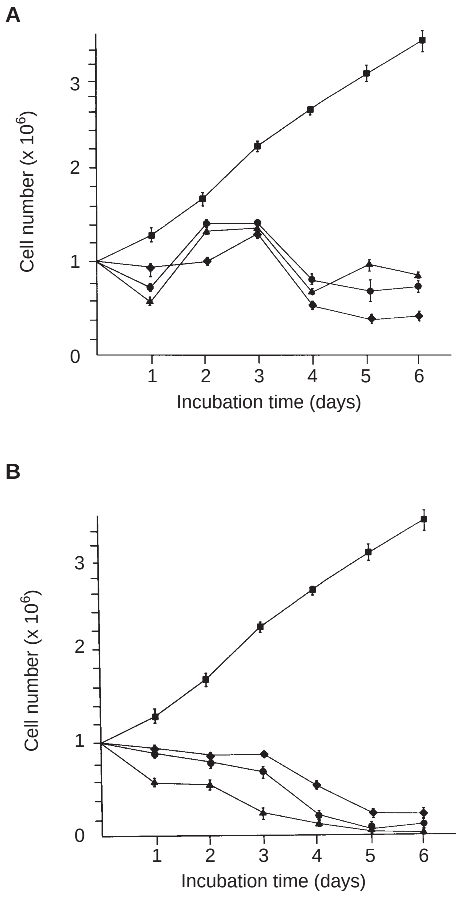

**Figure 2** Effect of treatment with GR-891 (**A**) and 5-FU (**B**) on the proliferation of rhabdomyosarcoma (RD) cells. Dose–response growth curves of untreated RD cells (■) and RD cells treated with 22.5 μmol l⁻¹ (△), 45 μmol l⁻¹ (●) or 90 μmol l⁻¹ (◆) GR-891 and 5-FU respectively. Results are the means of three experiments conducted in triplicate; bars, s.e.

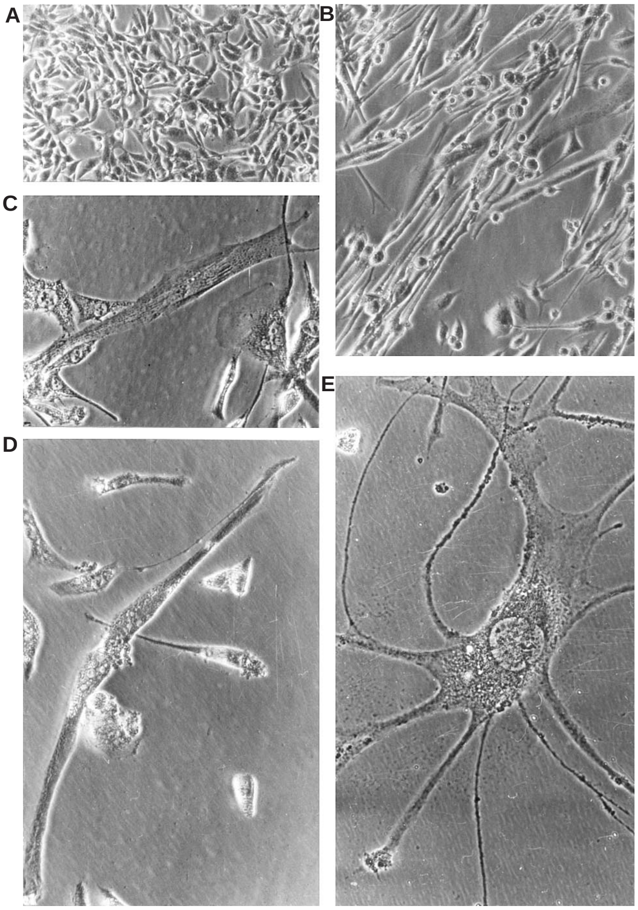

**Figure 3** Morphological changes caused by GR-891 in a human rhabdomyosarcoma cell line RD after 6 days of treatment. Phase-contrast microscopy of small spindle-shaped mononuclear cells under standard growth conditions. Typical cells of the RD parental cell line formed confluent aggregates, and grew in a monolayer (**A**) (× 6). After treatment with GR-891 (22.5 μmol l⁻¹), RD cells became elongated and grew in parallel (**B**) (× 6). These cells showed contact at points of the cell membrane and formed myotube-like giant cells (**C** and **D**) (× 12). Some cells occasionally developed many cytoplasmic projections which gave them an outline reminiscent of neuronal cells (**E**) (× 12)

final 10-min washing in PBS, the slides were mounted at pH 7.2 in FA mounting fluid (Difco Laboratories, Detroit, MI, USA). All cells were examined under a Nikon HFX-IIA light microscope for epifluorescence studies. Photographs were taken with a Nikon FX-35WA camera using Kodak TMAX 400 ASA paper.

### PCR evaluation of *mdr1* mRNA levels

Total RNA was obtained from untreated and treated RD cells according to Maniatis et al (1982) and reverse transcription was carried out with mdr1 or $\beta_2$-microglobulin ($\beta_2$m) primers as described by Nootan et al (1990). Levels of *mdr1* mRNA were estimated relative to $\beta_2$m mRNA using a modification of the RNA PCR method (Fuqua et al, 1990). The polymerase chain reaction (PCR) products obtained were loaded onto 2% agarose gels and visualized by ethidium bromide staining.

### Statistical analyses

The data derived from growth curves were subjected to analysis of variance with two independent factors. Student's *t*-test was used to analyse the differences between control and treated RD cells after FACS analysis. All data are means ± s.e.m. of three separate experiments, and a *P*-value less than or equal to 0.05 was considered significant.

### RESULTS

### Modification of growth rate by GR-891 and 5-FU

Treatment with GR-891 and 5-FU inhibited proliferation of the RD cell line. All concentrations of the drugs had a substantial inhibitory effect on growth during the first 3 days in comparison with the growth rate of control cells (Figure 2). After this period, proliferation was inhibited until day 6. Inhibition was significantly greater in cells treated with 22.5 μmol l⁻¹, 45 μmol l⁻¹ or 90 μmol l⁻¹ 5-FU than in cultures treated with different concentrations of GR-891. Cells treated once with GR-891 and 5-FU reached logarithmic growth appoximately 1 week after the drug was removed from the culture medium. Re-exposure of these logarithmically growing RD cells to drugs for an additional 6 days resulted in complete growth suppression that was no longer reversible after drugs were removed a

© Cancer Research Campaign 1999

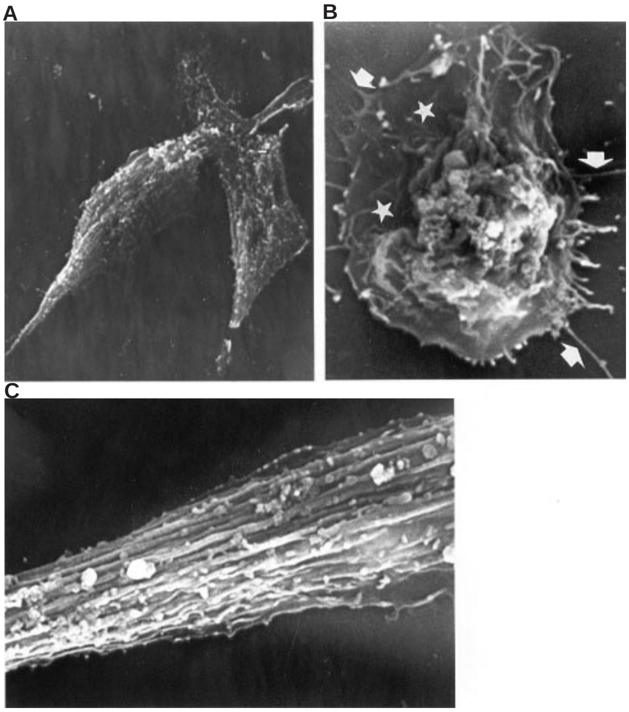

**Figure 4** Scanning electron micrograph images of untreated and treated RD cells for 6 days with 22.5 $\mu$mol l$^{-1}$ or 45 $\mu$mol l$^{-1}$ GR-891. Morphological characteristics of RD cells growing in standard medium (**A**) (× 1350). Membrane changes in RD cells treated with 45 $\mu$mol l$^{-1}$ GR-891. Note the appearance of phylopodia (thick arrows) and the attachment of cells to the substrate (stars) (**B**) (× 2100). Appearance of myotube-like giant cells showing myofibrils along the longitudinal axis (**C**) (× 3000) in RD cells treated with 22.5 $\mu$mol l$^{-1}$ GR-891

second time from the culture medium (data not shown). After treatment, the viability of cells in both control cultures and cultures treated with 22.5 $\mu$mol l$^{-1}$, 45 $\mu$mol l$^{-1}$ or 90 $\mu$mol l$^{-1}$ of GR-891 was >90%; however, there was a decrease in the percentage of viable cells to 73% in cultures treated with 5-FU.

### In vitro morphology

Observations with phase-contrast microscopy showed that parental RD cells had characteristics of undifferentiated cells (Figure 3A). During the first 2 days of treatment, cells growing with GR-891 and 5-FU showed no morphological changes in comparison with parental RD cells. However, after the 3rd day and until day 6, most of the cell population treated with 22.5 $\mu$mol l$^{-1}$ or 45 $\mu$mol l$^{-1}$ GR-891 grew in a whorl-like or a parallel pattern (Figure 3B), and showed an evident increase in the proportion of elongated and multinucleated cells (myotube-like giant cells) which represented up to 65% of the cell population (Figure 3C and D). Some cells developed many branching processes which gave them a characteristic neurite-like appearance (Figure 3E). In contrast, cell cultures treated with any dose of 5-FU showed few myotube-like giant cells (< 10%) and a larger proportion (82%) of dead cells after 6 days of treatment (not shown).

Examination of the parental RD cell line with SEM showed a variety of more or less elongated and polygonal shapes (Figure 4A). In contrast, the RD cells treated with 22.5 $\mu$mol l$^{-1}$ or 45 $\mu$mol l$^{-1}$ of GR-891 showed an evident increase in the cytoplasm, which adhered strongly to the substrate. These doses also increased the size and number of phylopodia and membrane folds (Figure 4B). Nevertheless, the most important characteristic of myogenic differentiation was the appearance of myotube-like giant cells in which the parallel arrangement of myofibrils along their longitudinal axis was evident (Figure 4C). In contrast, 5-FU led to the appearance of rounded cells with a very irregular contour and a surface split up into great portions which seem to show a current necrotic process (not shown).

RD cells treated for 6 days with GR-891 showed typical ultrastructural features of myogenic differentiation. In contrast to the RD parental cell line, treated cells had multiple nuclei consistent with giant cell formation, and the cytoplasm occupied large areas and contained increased numbers of oganelles including polyribosomes and rough endoplasmic reticulum (Figure 5A). A large number of glycogen deposits in clusters and elongated mitochondria with clear matrices and dilated cristae were visible. Treated cells contained many lipid vesicles throughout the cytoplasm and near the nucleus (Figure 5B), increased amounts of intermediate filaments (Figure 5C) and parallel myofilaments which tended to appear in well-defined bundles (Figure 5D) with electron-dense zones similar to Z-bands (Figure 5E). These changes were seen mainly in RD cells treated with 22.5 $\mu$mol l$^{-1}$ or 45 $\mu$mol l$^{-1}$ GR-891; similar features were seen in cells treated twice with these concentrations. In contrast, in cultures treated with 5-FU, few cells displayed these changes, and a larger proportion of cells showed characteristics of reduced cell viability (not shown).

### Modifications in intermediate filaments

Quantitative data on the changes in desmin and vimentin expression in cell line RD after 6 days of treatment with different concentrations of GR-891 and 5-FU were provided by FACS analysis (Table 1). Desmin and vimentin expression showed more evident changes in RD cells treated with 22.5 $\mu$mol l$^{-1}$ or 45 $\mu$mol l$^{-1}$ of GR-891 than with 90 $\mu$mol l$^{-1}$. There was a significant increase in

**Table 1** FACScan analysis of desmin and vimentin in the RD cell line after induction with GR-891 and 5-FU

| | Control | GR-891 ($\mu$M) | | | 5-FU ($\mu$M) |
|---|---|---|---|---|---|
| | | 22.5 | 45 | 90 | 90 |
| Desmin (%) | 29.93 ± 2.1 | 68.80 ± 1.6$^{a,c}$ | 63.04 ± 2.0$^{a,c}$ | 47.60 ± 1.8$^{b}$ | 48.52 ± 2.5$^{b}$ |
| Vimentin (%) | 86.30 ± 3.2 | 40.79 ± 2.4$^{a,c}$ | 57.51 ± 3.3$^{a,d}$ | 80.21 ± 2.2 | 72.56 ± 3.5 |

All data are means ± s.e.m. of four independent determinations. Significance was determined by comparison of the means with Student's *t*-test. [a]Significantly different ($P$ < 0.001) compared with parental RD. [b]Significantly different ($P$ < 0.005) compared with parental RD. [c]Significantly different ($P$ < 0.001) compared with RD cells treated with 5-FU. [d]Significantly different ($P$ < 0.005) compared with RD cells treated with 5-FU.

 *© Cancer Research Campaign 1999*

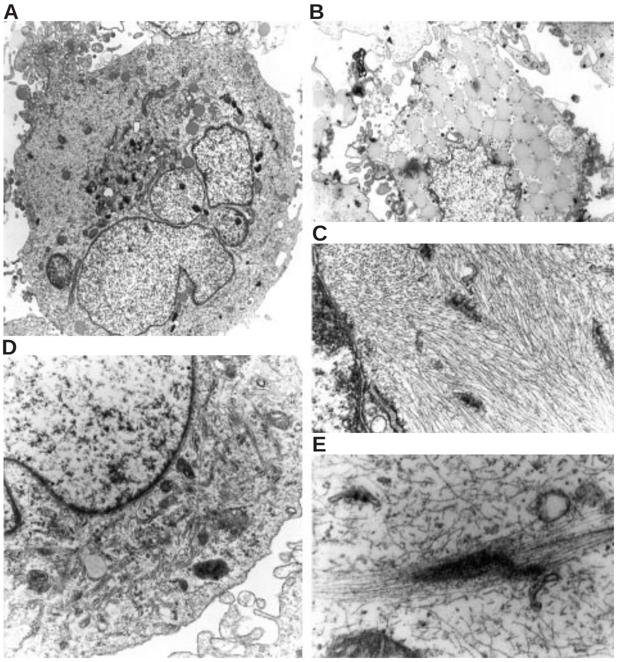

**Figure 5** Transmission electron micrographs of RD cells treated for 6 days with GR-891 (22.5 μmol l$^{-1}$). Multinucleated giant cell with nucleoli and increased numbers of cytoplasmic organelles (**A**) (× 2500). RD cells showing morphological changes of myogenic differentiation characterized by numerous lipid vesicles (**B**) (× 2500), highly organized intermediate filaments in the cytoplasm (**C**) (× 20 000) and abundant myofilament bundles (**D**) (× 8000) with zones similar to Z-bands (**E**) (× 32 500)

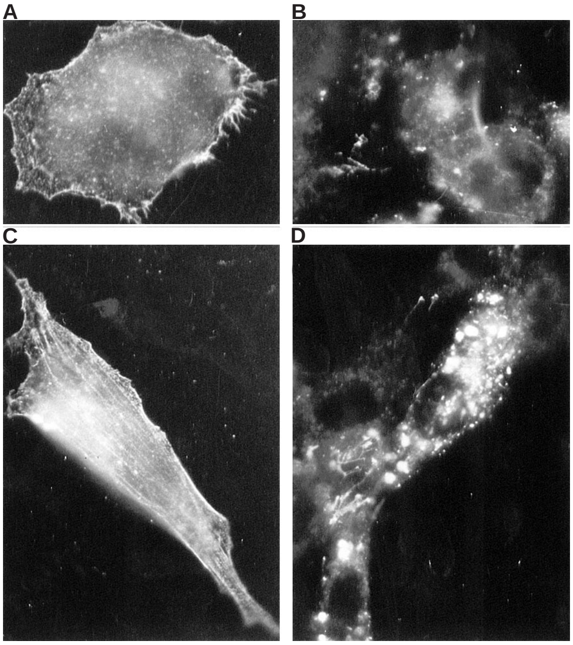

**Figure 6** Immunofluorescence micrographs of untreated and GR-891-treated RD cells for 6 days with 22.5 μmol l$^{-1}$. Low expression of α-actinin (**A**) (× 50) and fibronectin (**B**) (× 50) in RD cells growing under standard conditions. Treated RD cells showing intense labelling for α-actinin along the longitudinal axis of the cell (**C**) (× 50), and for fibronectin. Adhesion plaques desmin expression in RD cells treated with the lowest concentrations; however, only 29.93% of the parental RD cells expressed desmin. In comparison with the parental cell line, the percentage of vimentin-positive cells decreased significantly after treatment with 22.5 μmol l$^{-1}$ or 45 μmol l$^{-1}$ GR-891, whereas no significant change was found in RD cells growing in the presence of 90 μmol l$^{-1}$ of GR-891. RD cells treated with 90 μmol l$^{-1}$ 5-FU showed a significant increase in the labelling for desmin in relation to control RD cells. In contrast, no modifications were found in vimentin expression (Table 1). With doses of 22.5 μmol l$^{-1}$ or 45 μmol l$^{-1}$ 5-FU, we also found increased expression of desmin but no change in vimentin (data not shown).

### Immunofluorescent cytochemistry

Indirect immunofluorescence studies showed that the parental RD cell line expressed α-actinin in the plasma membrane, and scattered throughout the interior of the cells (Figure 6A). The expression of fibronectin was practically undetectable: in the cells in which this appeared, it was unevenly distributed as small round plaques throughout the cytoplasm and cellular membrane (Figure 6B).

Cells induced with the lowest concentrations of GR-891 showed intense labelling for α-actinin, which was well organized along phylopodia and the cytoplasmatic prolongations of myotube-like giant cells. Moreover, this protein appeared in the cytoplasm as parallel rows of immunofluorescent dots along the longitudinal axis of the cells (Figure 6C). The 90 μmol l$^{-1}$ concentration of both GR-891 and all doses of 5-FU led to slight modifications in the expression and distribution of the α-actinin, with more intense labelling than in the non-treated cells (not shown).

In cells induced with GR-891 and 5-FU, both the intensity of labelling in most cells and the size of the adhesion plaques were greater than in control cells. Adhesion plaques were distributed throughout the length of the cells, and in more differentiated cells we observed incipient extracellular expression on the rim of the cytoplasmatic prolongations. These changes were more evident in cells induced with the lowest concentrations of GR-891 (Figure 6D).

### Determination of *mdr1* expression by reverse transcription polymerase chain reaction (RT-PCR)

The parental RD cell line yielded a weak PCR product for *mdr1* (Figure 7). After induction for 6 days with any of the drug doses, there were no modifications in the PCR product for *mdr1* in the parental RD cell line. To demonstrate the integrity of the RNA preparations, PCR was performed using $\beta_2$m primers, and found to be of the same intensity. PCR amplification of $\beta_2$m demonstrated that the number of transcripts in the parental RD cell line and in RD cells treated with GR-891 and 5-FU was not the result of RNA degradation.

### DISCUSSION

New anti-cancer pyrimidine acyclonucleoside-like compounds synthesized in our laboratory (Campos et al, 1996; Gómez et al, 1997) act as prodrugs of 5-FU, and can be phosphorylated by the corresponding kinases and subsequently incorporated into RNA. GR-891 can release other substances with anti-tumour activity such as acrolein (Farquhar et al, 1995) and have low acute and chronic toxicity in vivo (Campos et al, 1996).

© Cancer Research Campaign 1999 

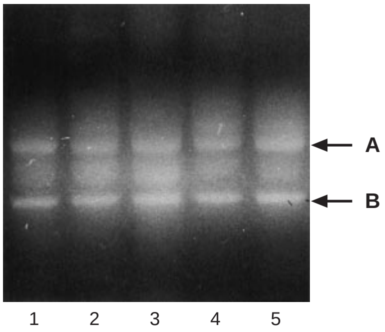

**Figure 7** Reverse transcriptase PCR of *mdr1* mRNA expression in RD cells (**A**) and $\beta_2$m mRNA determined as a control for the PCR technique (**B**). Lane 1, untreated RD cells; lane 2, RD cells treated with 90 μmol $l^{-1}$ 5-FU; lanes 3–5, RD cells treated with GR-891 at a dose of 22.5 μmol $l^{-1}$, 45 μmol $l^{-1}$ or 90 μmol $l^{-1}$ respectively

The results presented here demonstrate that GR-891 is able to induce myogenic differentiation in rhabdomyosarcoma cell cultures, in which normal differentiation is presumably interrupted resulting in uncontrolled cell growth and a malignant phenotype (Hazelton et al, 1987). This action on rhabdomyosarcoma differentiation has been recently shown by us using other 5-FU acyclonucleosides (Gómez et al 1997).

Rhabdomyosarcoma cells treated with 5-FU showed evident growth inhibition after 48 h, and decreased cell viability for as long as 6 days after treatment. This can be explained by the cytotoxic effect of 5-FU on this type of tumour, as was previously shown for other cell lines (Zupi et al, 1988; Waxman et al, 1990). In contrast, growth inhibition caused by 22.5 μmol $l^{-1}$ or 45 μmol $l^{-1}$ GR-891 was less marked and appeared later; cell survival after 6 days was 90%. Rhabdomyosarcoma cells treated with these doses become phenotypically more mature, and show characteristics of adult muscle cells (Gundersen et al, 1989) that indicate unequivocal features of myogenic differentiation as has been shown with other chemotherapeutic agents (Crouch et al, 1993; Melguizo et al, 1995; Marchal et al, 1997). The proportion of elongated and multinucleated giant cells increased, and these cells formed myotube-like giant cells. These structures contained features suggestive of myofibrils: scanning electron microscope images showed parallel bands which protruded along the longitudinal axis of the cell membrane. Ultrastructural changes included the appearance of an intense network of myofilaments arranged in structures reminiscent of Z-bands.

Morphological changes in RD cells were accompanied by modifications in the expression of differentiation markers (Carter et al, 1989; Prados et al, 1993; Wijnaendts et al, 1994). Treatment with lower doses of GR-891 modified the expression of desmin, vimentin and α-actinin, leading to a greater degree of organization of myofilaments in treated cells with a gradual substitution of vimentin by desmin and a uniform, linear distribution of α-actinin filaments along the actin myofilaments. These changes suggest that RD cells had re-entered the programme of muscle maturation and subsequently underwent a process similar to myogenesis (Burridge and Feramisco, 1981; Capetanaki et al, 1984; Wijnaendts et al, 1994). Minor changes in the degree of protein organization were observed with 5-FU, suggesting that its cytotoxic effect (Waxman et al, 1990, 1992; Lesuffleur et al, 1991*a*,*b*) hindered complete differentiation.

Because cells that had grown in the absence of the drug after 3 days of treatment had a proliferative rate similar to that of untreated cells and showed no morphological changes, our results suggest an active induction of differentiation, as has been proposed for other drugs (Chen and Breitman, 1994; D'Amore et al, 1994; Marchal et al, 1997), rather than selective destruction of undifferentiated cells. Moreover, within the parental cell population, we found no pre-existing differentiated population that might have indicated selective destruction.

The process of myogenic differentiation induced in the RD cell line by GR-891 was accompanied by increases in the expression, distribution and size of fibronectin plates. This protein of fibronexus is essential for the intercellular junction between myoblasts (Eyden, 1993), and takes part in the early phases of alignment and fusion of muscle cells (Le Moigne et al, 1990; Melo et al, 1996) and in subsequent morphological and structural changes during differentiation (Volk et al, 1990; Enomoto et al, 1993). Cell-substrate contacts and cell–cell interactions depend on the expression of fibronectin in rhabdomyosarcoma cells; both of these phenomena are related to the stage of maturation (Vogel et al, 1991) and with metastatic capacity (Langbein et al, 1990). The increase in fibronectin expression induced in RD cells by GR-891 suggests an increased degree of differentiation and a loss of metastatic potential. This supports the hypothetical inverse relationship between cancer malignancy and the degree of tumour differentiation. In rhabdomyosarcomas, Herrera et al (1995) showed that more differentiated cell lines were non-invasive and non-metastatic.

GR-891 did not increase the expression of the mdr1 gene, in contrast with other agents classically used to treat rhabdomyosarcomas. Although other drugs can increase the degree of cell differentiation, they also lead to mdr1 overexpression (Prados et al, 1996, 1998). Our findings indicate that GR-891 does not induce resistance, at least not via increased mdr1 expression. This represents an important potential advantage over other chemotherapeutic agents used to treat this type of tumour. Classic chemotherapeutic agents induce the expression of P-glycoprotein, which reduces the levels of the drug in the cell, and thus interferes with the effectiveness of these agents in inducing re-entry into the normal differentiation pathway (D'Amore et al, 1994; Melguizo et al, 1995).

In conclusion, we evaluate the potential role of GR-891, a new pyrimidine acyclonucleoside-like compound, for differentiation therapy against rhabdomyosarcomas. Our results in rhabdomyosarcoma cell cultures show that this new 5-FU prodrug inhibits cellular proliferation, induces morphological and ultrastructural changes typical of myogenic differentiation, and has limited cytotoxic effects. The increase in fibronectin expression suggests a decrease in the metastatic capacity of rhabdomyosarcoma cells. The low toxicity of GR-891 and the fact that it does not induce the MDR phenotype are significant advantages in comparison with 5-FU and other agents used in tumour differentiation therapy.

### ACKNOWLEDGEMENTS

This study was supported by the Comisión Interministerial de Ciencia y Tecnología (project QFN95-470) and by the Fondo de Investigación Sanitaria de la Seguridad Social (FIS) through project no. 95/0974 and BAE 97/5436 from JAM. We thank Karen Shashok for revising the English translation of the manuscript.

© Cancer Research Campaign 1999

### REFERENCES

Aguanno S, Bouche M, Adamo S and Molinaro M (1990) 12-*O*-Tetradecanoylphorbol-13-acetate-induced differentiation of a human rhabdomyosarcoma cell line. *Cancer Res* **50**: 3377–3382

Beere HM and Hickman JA (1993) Differentiation: a suitable strategy for cancer chemotherapy? *Anticancer Drug Dev* **8**: 299–322

Borden EC, Lotan R, Levens D, Young CW and Waxman S (1993) Differentiation therapy of cancer: laboratory and clinical investigations. *Cancer Res* **53**: 4109–4115

Burridge K and Feramisco JR (1981) Non-muscle α-actinins are calcium-sensitive actin binding proteins. *Nature* **294**: 565–567

Campos J, Pineda MJ, Gómez JA, Entrena A, Trujillo MA, Gallo MA and Espinosa A (1996) 5-fluorouracil derivatives. 1. Acyclonucleosides through a tin (IV) chloride-mediated regiospecific ring opening of alkoxy-1,4-diheteroepanes. *Tetrahedron* **52**: 8907–8924

Capetanaki YG, Ngai J and Lazarides E (1984) Characterization and regulation in the expression of a gene coding for the intermediate filament desmin. *Proc Natl Acad Sci USA* **81**: 6909–6913

Carter RL, McCarthy KP, Machin LG, Jameson CF, Philip ER and Pinkerton CR (1989) Expression of desmin and myoglobin in rhabdomyosarcomas and developing skeletal muscle. *Histopathology* **1**: 585–595

Chen ZX and Breitman TR (1994) Tributyrin: a prodrug of butyric acid for potential clinical application in differentiation therapy. *Cancer Res* **54**: 3494–3499

Crouch GD and Helman LJ (1991) All-*trans*-retinoic acid inhibits the growth of human rhabdomyosarcoma cell lines. *Cancer Res* **51**: 4882–4887

Crouch GD, Kalebic T, Tsokos M and Helman LJ (1993) Ara-C treatment leads to differentiation and reverses the transformed phenotype in a human rhabdomyosarcoma cell line. *Exp Cell Res* **204**: 210–216

D'Amore ESG, Tollot M, Stracca-Pansa V, Menegon A, Mell S and Ninfo V (1994) Therapy associated differentiation in rhabdomyosarcomas. *Modern Pathol* **7**: 69–75

De Giovanni C, Lollini PL, Dolcetti R, Landuzzi L, Nicpletti G, D'Andrea E, Scotland K and Nanni P (1993) Uncoupling of growth inhibition and differentiation in dexamethasone-treated human rhabdomyosarcoma cells. *Br J Cancer* **67**: 674–679

Degos L (1995) A new concept: treatment using differentiation of the malignant cell in man. *Bull Acad Natl Med* **179**: 1689–1700

Enomoto MI, Boettiger D and Menko AS (1993) Alpha 5 integrin is a critical component of adhesion plaques in myogenesis. *Dev Biol* **155**: 180–197

Eyden BP (1993) Brief review of the fibronexus and its significance for myofibroblastic differentiation and tumor diagnosis. *Ultrastruct Pathol* **17**: 611–622

Farquhar D, Chen R and Khan S (1995) 5′-[4-(Pivaloyloxy)-1,3,2-dioxaphosphorinan-2-yl]-2′-deoxy-5-fluorouridine: a membrane-permeating prodrug of 5-fluoro-2′-deoxyuridylic acid (FdUMP). *J Med Chem* **38**: 488–495

Fuqua SA, Fitzgerald SD and McGuire WL (1990) A simple polymerase chain reaction method for detection and cloning of low-abundance transcripts. *Biotechniques* **9**: 206–211

Gabbert HE, Gerharz CD, Biesalsk HK, Engers R and Luley C (1988) Terminal differentiation and growth inhibition of a rat rhabdomyosarcoma cell line (BA-HAN-1C) in vitro after exposure to retinoic acid. *Cancer Res* **48**: 5264–5269

Germann UA (1996) P-glycoprotein: a mediator of multidrug resistance in tumour cells. *Eur J Cancer* **32**: 927–944

Gómez JA, Campos J, Marchal JA, Trujillo MA, Melguizo C, Prados J, Gallo MA, Aránega A and Espinosa A (1997) Chemical modifications on the acyclic moiety of 3-(2-hydroxyethoxy)-1-alkoxypropyl nucleobases. 2. Differentiation and growth inhibition in rhabdomyosarcoma cells after exposure to a novel 5-fluorouracil acyclonucleoside. *Tetrahedron* **53**: 7319–7334

Gundersen GG, Khawaja S and Bulinski JC (1989) Generation of a stable, post-translationally modified microtubule array is an early event in myogenic differentiation. *J Cell Biol* **109**: 2275–2288

Hazelton BJ, Houghton JA, Parham DM, Douglass EL, Torrance PM, Holt H and Houghton PJ (1987) Characterization of cell lines derived from xenographs of childhood rhabdomyosarcoma. *Cancer Res* **47**: 4501–4507

Herrera A, Royal A and Babai F (1995) Correlation between cell differentiation, types of invasion, and hematogenous metastasis in experimental rhabdomyosarcomas. *Exp Mol Pathol* **63**: 1–15

Langbein L, Kosmehl H, Katenkamp D, Neupert G and Stiller KJ (1990) Experimentally induced murine rhabdomyosarcomas – correlation between cellular contacts, matrix formation and cellular differentiation. *Differentiation* **44**: 185–196

Le Moigne A, Martelly I, Barlovatz G, Franquinet R, Aamiri A, Frisdal E, Bassaglia Y, Moraczewski G and Gautron J (1990) Characterization of myogenesis from adult satellite cells cultured in vitro. *Int J Dev Biol* **34**: 171–180

Lesuffleur T, Kornowski A, Augeron C, Dussaulx E, Barbat A, Laboisse C and Zweibaum A (1991*a*) Increased growth adaptability to 5-fluorouracil and methotrexate of HT-29 subpopulations selected for their commitment to differentiation. *Int J Cancer* **49**: 731–737

Lesuffleur T, Kornowski A, Luccioni C, Muleris M, Barbat A, Beaumatin J, Dussaulx E, Dutrillaux B and Zweibaum A (1991*b*) Adaptation to 5-fluorouracil of the heterogeneous human colon tumor cell line HT-29 results in the selection of cells committed to differentiation. *Int J Cancer* **49**: 721–730

Lollini PL, De Giovanni C, Del Re B, Landuzzi L, Nicoletti G and Prodi G (1989) Myogenic differentiation of human rhabdomyosarcoma cells induced in vitro by antineoplastic drugs. *Cancer Res* **49**: 3631–3636

MacAllister RM, Melnyk J, Finklestein JZ, Adams EC and Gardner MB (1969) Cultivation in vitro of cells derived from a human rhabdomyosarcoma. *Cancer* **24**: 520–526

Maniatis T, Fristch E and Sambrook J (1982) Molecular cloning. In *Cold Spring Harbor, A Laboratory Manual*. Cold Spring Harbor Laboratory: New York

Marchal JA, Prados J, Melguizo C, Fernández JE, Vélez C, Alvarez L and Aránega A (1997) Actinomycin D treatment leads to differentiation and inhibits proliferation in rhabdomyosarcoma cells. *J Lab Clin Med* **130**: 42–50

Melguizo C, Prados J, Aneiros J, Fernández JE, Vélez C and Aránega A (1995) Differentiation of a human rhabdomyosarcoma cell line after antineoplastic drug treatment. *J Pathol* **175**: 23–29

Melguizo C, Prados J, Marchal JA, Aránega AE, Alvarez L and Aránega A (1996) Low concentrations of actinomycin D potentially cause therapeutic differentiation in human rhabdomyosarcoma cell line RD. *Path. Res Pract* **192**: 188–194

Melo F, Carey DJ and Brandan E (1996) Extracellular matrix is required for skeletal muscle differentiation but not myogenin expression. *J Cell Biochem* **62**: 227–239

Nootan KE, Beck C, Holzmayer TA, Chin JE, Wunder JS, Andrulis IL, Gazdar AF, Willman CL, Griffith B, Von Hoff DD and Roninson IB (1990) Quantitative analysis of *MDR1* (multidrug resistance) gene expression in human tumors by polymerase chain reaction. *Proc Natl Acad Sci USA* **87**: 7160–7164

Prados J, Melguizo C, Fernández JE, Aránega AE, Alvarez L and Aránega A (1993) Actin, tropomyosin and α-actinin as markers of differentiation in human rhabdomyosarcoma cell lines induced with dimethyl sulfoxide. *Cell Mol Biol* **39**: 525–536

Prados J, Melguizo C, Fernández JE, Aránega AE, Alvarez L and Aránega A (1996) Inverse expression of *mdr1* and c-*myc* genes in a rhabdomyosarcoma cell line resistant to actinomycin D. *J Pathol* **180**: 85–89

Prados J, Melguizo C, Marchal JA, Vélez C, Alvarez L and Aránega A (1998) Therapeutic differentiation in a human rhabdomyosarcoma cell line selected for resistance to actinomycin D. *Int J Cancer* **75**: 379–383

Sartorelli A (1985) Malignant cell differentiation as a potential therapeutic approach. *Br J Cancer* **52**: 293–302

Skach WR (1996) Transmembrane orientation and topogenesis of the third and fourth membrane-spanning regions of human P-glycoprotein (MDR1). *Cancer Res* **54**: 3202–3209

Vogel W, Kosmehl H, Katenkamp D and Langbein L (1991) Differentiation dependent matrix formation (fibronectin and laminin) in an experimental murine rhabdomyosarcoma model. *Acta Histochem* **90**: 181–188

Volk T, Fessler LI and Fessler JH (1990) A role for integrin in the formation of sarcomeric cytoarchitecture. *Cell* **63**: 525–536

Waxman S, Scher BM, Hellinger N and Scher W (1990) Combination cytotoxic-differentiation therapy of mouse erythroleukemia cells with 5-fluorouracil and hexamethylene bisacetamide. *Cancer Res* **50**: 3878–3887

Waxman S, Huang Y, Scher BM and Scher M (1992) Enhancement of differentiation and cytotoxicity of leukemia cells by combinations of fluorinated pyrimidines and differentiation inducers: development of DNA double-strand breaks. *Biomed Pharmacother* **46**: 183–192

Wijnaendts LCD, Van Der Linden JC, Van Unnik AJM, Delemarre JFM, Voute PA and Meijer CJL (1994) The expression pattern of contractile and intermediate filament proteins in developing skeletal muscle and rhabdomyosarcoma of childhood: diagnostic and prognostic utility. *J Pathol* **174**: 283–292

Zupi G, Marangolo M, Arancia G, Greco C, Laudonio N, Iosi F, Formisano G and Malorni W (1988) Modulation of the cytotoxic effect of 5-fluorouracil by *N*-methylformamide on a human colon carcinoma cell line. *Cancer Res* **48**: 6193–6200

© Cancer Research Campaign 1999

## References

[PDF_00533] Aguanno S., Bouchè M., Adamo S., Molinaro M. (1990). 12-O-tetradecanoylphorbol-13-acetate-induced differentiation of a human rhabdomyosarcoma cell line.. Cancer Res.

[PDF_00536] Beere H. M., Hickman J. A. (1993). Differentiation: a suitable strategy for cancer chemotherapy?. Anticancer Drug Des.

[PDF_00538] Borden E. C., Lotan R., Levens D., Young C. W., Waxman S. (1993). Differentiation therapy of cancer: laboratory and clinical investigations.. Cancer Res.

[PDF_00541] Burridge K., Feramisco J. R. (1981). Non-muscle alpha actinins are calcium-sensitive actin-binding proteins.. Nature.

[PDF_00547] Capetanaki Y. G., Ngai J., Lazarides E. (1984). Characterization and regulation in the expression of a gene coding for the intermediate filament protein desmin.. Proc Natl Acad Sci U S A.

[PDF_00550] Carter R. L., McCarthy K. P., Machin L. G., Jameson C. F., Philp E. R., Pinkerton C. R. (1989). Expression of desmin and myoglobin in rhabdomyosarcomas and in developing skeletal muscle.. Histopathology.

[PDF_00553] Chen Z. X., Breitman T. R. (1994). Tributyrin: a prodrug of butyric acid for potential clinical application in differentiation therapy.. Cancer Res.

[PDF_00555] Crouch G. D., Helman L. J. (1991). All-trans-retinoic acid inhibits the growth of human rhabdomyosarcoma cell lines.. Cancer Res.

[PDF_00557] Crouch G. D., Kalebic T., Tsokos M., Helman L. J. (1993). Ara-C treatment leads to differentiation and reverses the transformed phenotype in a human rhabdomyosarcoma cell line.. Exp Cell Res.

[PDF_00563] De Giovanni C., Lollini P. L., Dolcetti R., Landuzzi L., Nicoletti G., D'Andrea E., Scotland K., Nanni P. (1993). Uncoupling of growth inhibition and differentiation in dexamethasone-treated human rhabdomyosarcoma cells.. Br J Cancer.

[PDF_00567] Degos L. (1995). Un concept nouveau: le traitement par différenciation de la cellule maligne de l'homme.. Bull Acad Natl Med.

[PDF_00569] Enomoto M. I., Boettiger D., Menko A. S. (1993). Alpha 5 integrin is a critical component of adhesion plaques in myogenesis.. Dev Biol.

[PDF_00571] Eyden B. P. (1993). Brief review of the fibronexus and its significance for myofibroblastic differentiation and tumor diagnosis.. Ultrastruct Pathol.

[PDF_00574] Farquhar D., Chen R., Khan S. (1995). 5'-[4-(Pivaloyloxy)-1,3,2-dioxaphosphorinan-2-yl]-2'-deoxy-5-fluorouridine: a membrane-permeating prodrug of 5-fluoro-2'-deoxyuridylic acid (FdUMP).. J Med Chem.

[PDF_00577] Fuqua S. A., Fitzgerald S. D., McGuire W. L. (1990). A simple polymerase chain reaction method for detection and cloning of low-abundance transcripts.. Biotechniques.

[PDF_00580] Gabbert H. E., Gerharz C. D., Biesalski H. K., Engers R., Luley C. (1988). Terminal differentiation and growth inhibition of a rat rhabdomyosarcoma cell line (BA-HAN-1C) in vitro after exposure to retinoic acid.. Cancer Res.

[PDF_00583] Germann U. A. (1996). P-glycoprotein--a mediator of multidrug resistance in tumour cells.. Eur J Cancer.

[PDF_00590] Gundersen G. G., Khawaja S., Bulinski J. C. (1989). Generation of a stable, posttranslationally modified microtubule array is an early event in myogenic differentiation.. J Cell Biol.

[PDF_00593] Hazelton B. J., Houghton J. A., Parham D. M., Douglass E. C., Torrance P. M., Holt H., Houghton P. J. (1987). Characterization of cell lines derived from xenografts of childhood rhabdomyosarcoma.. Cancer Res.

[PDF_00596] Herrera-Gayol A., Royal A., Babaï F. (1995). Correlation between cell differentiation stage, types of invasion, and hematogenous metastasis in experimental rhabdomyosarcomas.. Exp Mol Pathol.

[PDF_00599] Langbein L., Kosmehl H., Katenkamp D., Neupert G., Stiller K. J. (1990). Experimentally induced murine rhabdomyosarcomas--correlation between cellular contacts, matrix formation and cellular differentiation.. Differentiation.

[PDF_00603] Le Moigne A., Martelly I., Barlovatz-Meimon G., Franquinet R., Aamiri A., Frisdal E., Bassaglia Y., Moraczewski G., Gautron J. (1990). Characterization of myogenesis from adult satellite cells cultured in vitro.. Int J Dev Biol.

[PDF_00606] Lesuffleur T., Kornowski A., Augeron C., Dussaulx E., Barbat A., Laboisse C., Zweibaum A. (1991). Increased growth adaptability to 5-fluorouracil and methotrexate of HT-29 sub-populations selected for their commitment to differentiation.. Int J Cancer.

[PDF_00610] Lesuffleur T., Kornowski A., Luccioni C., Muleris M., Barbat A., Beaumatin J., Dussaulx E., Dutrillaux B., Zweibaum A. (1991). Adaptation to 5-fluorouracil of the heterogeneous human colon tumor cell line HT-29 results in the selection of cells committed to differentiation.. Int J Cancer.

[PDF_00614] Lollini P. L., De Giovanni C., Del Re B., Landuzzi L., Nicoletti G., Prodi G., Scotlandi K., Nanni P. (1989). Myogenic differentiation of human rhabdomyosarcoma cells induced in vitro by antineoplastic drugs.. Cancer Res.

[PDF_00622] Marchal J. A., Prados J., Melguizo C., Fernández J. E., Vélez C., Alvarez L., Aránega A. (1997). Actinomycin D treatment leads to differentiation and inhibits proliferation in rhabdomyosarcoma cells.. J Lab Clin Med.

[PDF_00617] McAllister R. M., Melnyk J., Finkelstein J. Z., Adams E. C., Gardner M. B. (1969). Cultivation in vitro of cells derived from a human rhabdomyosarcoma.. Cancer.

[PDF_00625] Melguizo C., Prados J., Aneiros J., Fernandez J. E., Velez C., Aranega A. (1995). Differentiation of a human rhabdomyosarcoma cell line after antineoplastic drug treatment.. J Pathol.

[PDF_00628] Melguizo C., Prados J., Marchal J. A., Aránega A. E., Alvarez L., Aránega A. (1996). Low concentrations of actinomycin D potentially cause therapeutic differentiation in human rhabdomyosarcoma cell line RD.. Pathol Res Pract.

[PDF_00632] Melo F., Carey D. J., Brandan E. (1996). Extracellular matrix is required for skeletal muscle differentiation but not myogenin expression.. J Cell Biochem.

[PDF_00635] Noonan K. E., Beck C., Holzmayer T. A., Chin J. E., Wunder J. S., Andrulis I. L., Gazdar A. F., Willman C. L., Griffith B., Von Hoff D. D. (1990). Quantitative analysis of MDR1 (multidrug resistance) gene expression in human tumors by polymerase chain reaction.. Proc Natl Acad Sci U S A.

[PDF_00639] Prados J., Melguizo C., Fernandez J. E., Aranega A. E., Alvarez L., Aranega A. (1993). Actin, tropomyosin and alpha-actinin as markers of differentiation in human rhabdomyosarcoma cell lines induced with dimethyl sulfoxide.. Cell Mol Biol (Noisy-le-grand).

[PDF_00643] Prados J., Melguizo C., Fernández A., Aránega A. E., Alvarez L., Aránega A. (1996). Inverse expression of mdr 1 and c-myc genes in a rhabdomyosarcoma cell line resistant to actinomycin d.. J Pathol.

[PDF_00646] Prados J., Melguizo C., Marchal J. A., Vélez C., Alvarez L., Aránega A. (1998). Therapeutic differentiation in a human rhabdomyosarcoma cell line selected for resistance to actinomycin D.. Int J Cancer.

[PDF_00649] Sartorelli A. C. (1985). The 1985 Walter Hubert lecture. Malignant cell differentiation as a potential therapeutic approach.. Br J Cancer.

[PDF_00651] Skach W. R., Lingappa V. R. (1994). Transmembrane orientation and topogenesis of the third and fourth membrane-spanning regions of human P-glycoprotein (MDR1).. Cancer Res.

[PDF_00654] Vogel W., Kosmehl H., Katenkamp D., Langbein L. (1991). Differentiation dependent matrix formation (fibronectin and laminin) in an experimental murine rhabdomyosarcoma model.. Acta Histochem.

[PDF_00657] Volk T., Fessler L. I., Fessler J. H. (1990). A role for integrin in the formation of sarcomeric cytoarchitecture.. Cell.

[PDF_00662] Waxman S., Huang Y., Scher B. M., Scher M. (1992). Enhancement of differentiation and cytotoxicity of leukemia cells by combinations of fluorinated pyrimidines and differentiation inducers: development of DNA double-strand breaks.. Biomed Pharmacother.

[PDF_00659] Waxman S., Scher B. M., Hellinger N., Scher W. (1990). Combination cytotoxic-differentiation therapy of mouse erythroleukemia cells with 5-fluorouracil and hexamethylene bisacetamide.. Cancer Res.

[PDF_00666] Wijnaendts L. C., van der Linden J. C., van Unnik A. J., Delemarre J. F., Voute P. A., Meijer C. J. (1994). The expression pattern of contractile and intermediate filament proteins in developing skeletal muscle and rhabdomyosarcoma of childhood: diagnostic and prognostic utility.. J Pathol.

[PDF_00671] Zupi G., Marangolo M., Arancia G., Greco C., Laudonio N., Iosi F., Formisano G., Malorni W. (1988). Modulation of the cytotoxic effect of 5-fluorouracil by N-methylformamide on a human colon carcinoma cell line.. Cancer Res.

[PDF_00560] d'Amore E. S., Tollot M., Stracca-Pansa V., Menegon A., Meli S., Carli M., Ninfo V. (1994). Therapy associated differentiation in rhabdomyosarcomas.. Mod Pathol.

